# Identification and prediction of immune checkpoint inhibitors-related pneumonitis by machine learning

**DOI:** 10.3389/fimmu.2023.1138489

**Published:** 2023-06-29

**Authors:** Li Gong, Jun Gong, Xin Sun, Lin Yu, Bin Liao, Xia Chen, Yong-sheng Li

**Affiliations:** ^1^ Department of Phase I Clinical Trial Ward, Chongqing Key Laboratory of Translational Research for Cancer Metastasis and Individualized Treatment, Chongqing University Cancer Hospital, Chongqing, China; ^2^ Department of Information Center, The University Town Hospital of Chongqing Medical University, Chongqing, China; ^3^ Department of Artificial Intelligence, NanPeng Artificial Intelligence Research Institute Ltd., Chongqing, China; ^4^ Clinical Research Center, Chongqing Key Laboratory of Translational Research for Cancer Metastasis and Individualized Treatment, Chongqing University Cancer Hospital, Chongqing, China

**Keywords:** immune checkpoint inhibitors, pneumonitis, risk prediction, machine learning, risk factors

## Abstract

**Background:**

Immune checkpoint inhibitor (ICI)-related pneumonitis (IRP) is a common and potentially fatal clinical adverse event. The identification and prediction of the risk of ICI-related IRP is a major clinical issue. The objective of this study was to apply a machine learning method to explore risk factors and establish a prediction model.

**Methods:**

We retrospectively analyzed 48 patients with IRP (IRP group) and 142 patients without IRP (control group) who were treated with ICIs. An Elastic Net model was constructed using a repeated k-fold cross-validation framework (repeat = 10; k = 3). The prediction models were validated internally and the final prediction model was built on the entire training set using hyperparameters with the best interval validation performance. The generalizability of the final prediction model was assessed by applying it to an independent test set. The overall performance, discrimination, and calibration of the prediction model were evaluated.

**Results:**

Eleven predictors were included in the final predictive model: sindillizumab, number of ≥2 underlying diseases, history of lung diseases, tirelizumab, non-small cell lung cancer (NSCLC), percentage of CD4^+^ lymphocytes, body temperature, KPS score ≤70, hemoglobin, cancer stage IV, and history of antitumor therapy. The external validation of the risk prediction model on an independent test set of 37 patients and showed good discrimination and acceptable calibration ability: with AUC of 0.81 (95% CI 0.58–0.90), AP of 0.76, scaled Brier score of 0.31, and Spiegelhalter-z of −0.29 (P-value:0.77). We also designed an online IRP risk calculator for use in clinical practice.

**Conclusion:**

The prediction model of ICI-related IRP provides a tool for accurately predicting the occurrence of IRP in patients with cancer who received ICIs.

## 1 Introduction

Immune checkpoint inhibitors (ICIs) are a new class of anticancer drugs that activate T-cell-mediated immune responses against tumor cells (1). Therapeutically blocking inhibitory molecules include cytotoxic T-lymphocyte antigen 4 (CTLA4) inhibitors, programmed cell death 1 (PD1) inhibitors, and programmed cell death 1 ligand (PD-L1) inhibitors (2). Trials have confirmed the clinical efficacy of ICIs in various advanced malignancies (2), and ICIs are emerging as a first-line treatment for some advanced cancers (2, 3). ICIs can result in a special set of adverse events termed immune-related adverse events (irAEs) (4, 5). IrAEs occur in all tissues and organs, most commonly in the lungs, skin, and liver. Common fatal irAEs are pneumonitis, myocarditis, colitis, hepatitis, and neurological effects (6, 7). Immune-related pneumonitis (IRP) is a clinically common, serious, and potentially lethal irAE, which develops in approximately 3.5%–19% of ICI therapy cases and accounts for 35% of ICI-related deaths (7). IRP can result in a high rate of treatment discontinuation (8) and cause a major economic burden on cancer patients (9). IRP is difficult to diagnose and there is no gold standard for clinical diagnosis (7).

ICI-related IRP requires significant attention given its clinical severity and diagnostic challenges (10). Previous studies have demonstrated that identification and prediction of the risk of ICI-related IRP are major issues (7, 11). Early prediction of the risk of IRP would reduce safety risks and improve clinical benefits. Establishing a prediction model is an effective way to achieve early prediction of the IRP. Machine learning is a new artificial intelligence method, which has been widely used to explore predictive factors and establish prediction models (12, 13). However, to date, no study has attempted to develop predictive models for IRP. In this study, we aimed to establish a prediction model to quantify individuals’ IRP risk and provide an IRP risk prediction online calculator for clinical practice.

## 2 Materials and methods

### 2.1 Study data

We extracted electronic medical records from the Scientific Research Data Platform of patients discharged from Chongqing University Cancer Hospital between 1 January 2010, and 31 December 2021. Two clinicians were assigned to review the extracted electronic medical records independently and determine each patient’s IRP status (IRP or non-IRP) and eligibility for this study. The diagnosis of IRP or non-IRP was based on the patient’s clinical symptoms, laboratory test results, and the physician’s clinical experience. Patients with a confirmed IRP diagnosis were classified into the IRP group. Patients who were diagnosed with pneumonitis but were not associated with an immune reaction or did not develop pneumonitis were classified into the non-IRP group. We first identified and included IRP cases, after which we randomly sampled non-IRP cases using a sample size four times the number of IRP cases. Patients were included in the IRP and non-IRP groups at a ratio of 1:4. The included patients were:1) aged 18 or above; 2) male and female; 3) diagnosed with cancer according to the pathological and clinical diagnosis; 4) treated with ICIs (only mono immunotherapy) in-hospital; and 5) never developed IRP before ICIs treatment. We excluded patients 1) whose treatment option was not ICIs and 2) patients receiving combination ICIs therapies.

This was a retrospective study and informed consent was not required. This study was approved by the Ethics Committee of the Chongqing University Cancer Hospital (CZLS2021042-A).

### 2.2 Study outcome and variables

The outcome of interest was IRP, defined as the manifestation of pneumonitis after ICI therapies related to immune reactions (14). Candidate predictors included the patients’ demographic information (sex, age, height, weight, etc., which were measured before the assignment of ICI treatment), body temperature (refers to the forehead temperature measured by an Infrared Thermometer or armpit temperature measured by a Mercury Thermometer, and we selected the most recent result prior to ICIs initiation), disease situation (cancer types and cancer stage, etc.), treatment information (ICI drugs type, ICI dosage, number of combined drugs, previous treatment, etc.), and laboratory test data (blood routine examination, inflammatory, arterial blood gas, etc.), which were collected from the most recent laboratory test performed after cancer treatment and before the onset of IRP.

A complete list of the variables is provided in detail in **Supplemental Appendix 1** **(** **Table S1** **)**. Variables with a missing rate less than 15% were included. The handling of missing data is described in detail in **Supplemental Appendix 2** (**Table S3**) and the preprocessing section in **Supplemental Appendix 3**.

### 2.3 Statistical analysis

All potential predictors were summarized and stratified according to the IRP status. Continuous and categorical variables were described as median (IQR) and frequency (percentage), respectively. Univariate analyses of each predictor between the IRP and non-IRP groups were conducted, continuous variables were assessed using the Kruskal–Wallis test, and categorical variables were analyzed using the chi-squared test or Fisher’s exact test, as appropriate. The median or mode was used to impute missing data. Stratified sampling was used to divide the working dataset into two parts at a ratio of 8:2 (called the training and test sets, respectively). Subsequently, a multivariable risk prediction model was developed on the training set using the Elastic Net under a repeated k-fold cross-validation (repeats = 10; k = 3) framework. Specifically, for each combination of hyperparameters, the training set was randomly partitioned into three roughly equal sized parts; one part was left as the validation set, and the model was built on the remaining parts. The leave-out modeling process was conducted recursively until each part was treated as a validation set. The cross-validation modeling process was repeated 10 times, and the performance was evaluated on 30 validation sets. This procedure was repeated using different hyperparameter settings (we tuned 100 combinations of hyperparameters; the values are provided in **Supplemental Appendix 3**). The final prediction model was built on the entire training set using hyperparameters that yielded the best internal validation performance. Furthermore, the final prediction model was applied to external data (i.e., the test set) for external validation. The detailed modeling process is provided in **Supplemental Appendix 3** (**Figure S2**).

**Figure 1:**
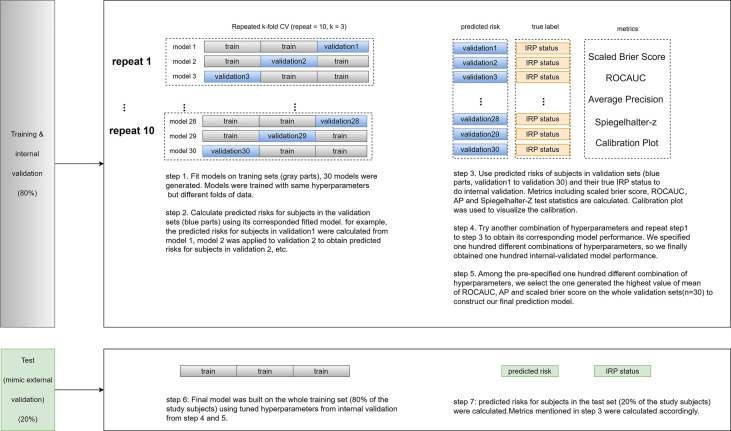
The workflow of the IRP prediction model building.

The overall performance of the model was evaluated using the scaled Brier scores (SBrSs). Model discrimination was assessed using the area under the ROC curve (AUC), whose 95% confidence interval was obtained using bootstrapping, and calibration was evaluated using average precision (AP, the area under the precision-recall curve) and Spiegelhalter-z statistics. SHAP (Shapley Additive exPlanations) values was utilized to visualize the variable importance. Calibration plots and risk stratification results were generated to examine model performance in different sub-risk groups. An online calculator was developed using R shiny, which allows clinicians and cancer survivors to calculate personalized IRP risks. All performance matrices were computed on the validation and test sets, and the metrics reported in the *Results* section were cross-validated (**Figure 1**).

Statistical analyses were conducted in R (version 4.1.2) and *P-*values less than 0.05 were considered statistically significant, and all tests were two-tailed.

The code generating all results is publicly available [https://github.com/gongli0707/IRP-prediction].

## 3 Results

### 3.1 Study patients

**Figure 2:**
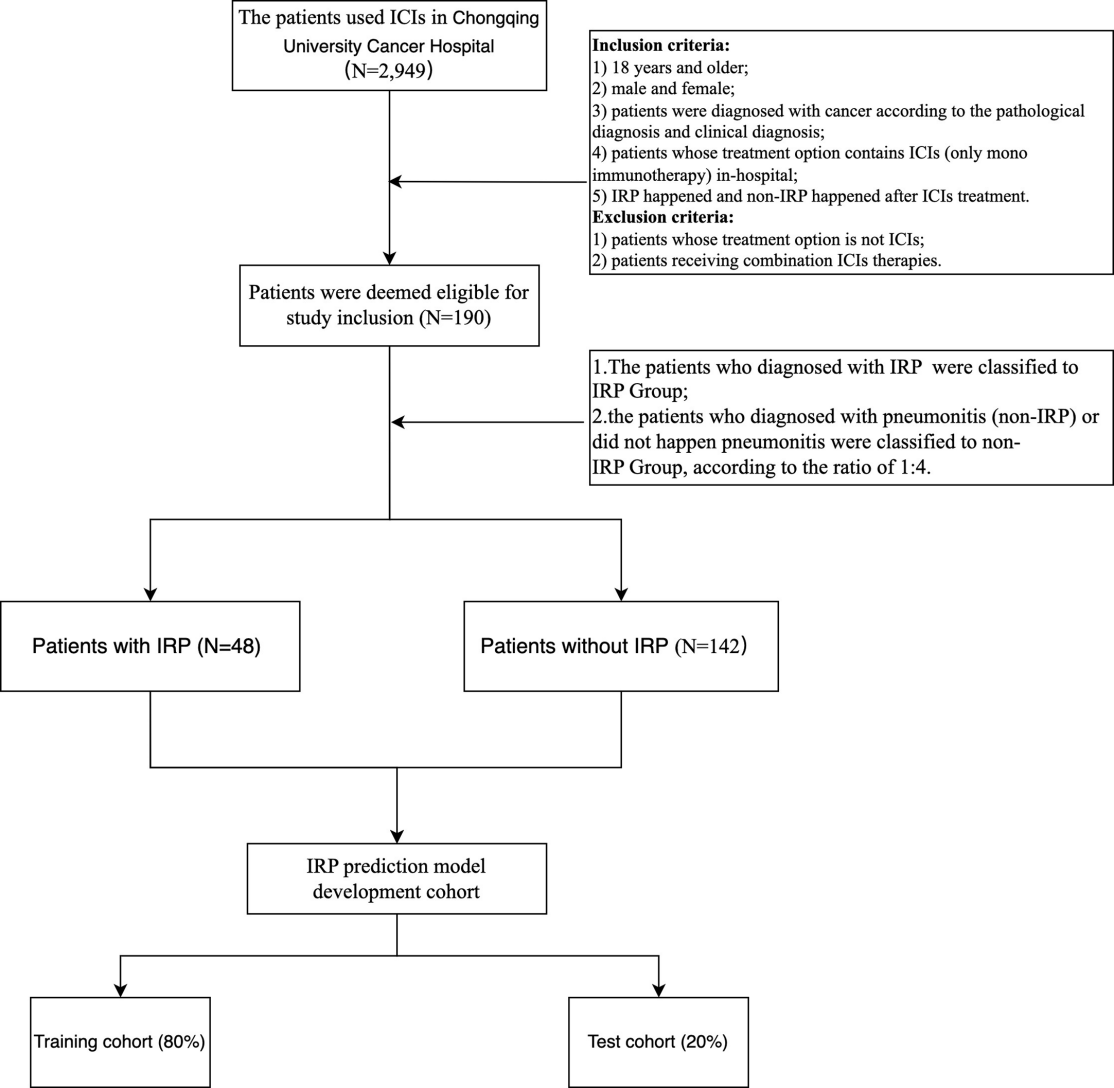
The flow chart of this study.

According to the inclusion and exclusion criteria, 190 cases were identified from the Scientific Research Data Platform of Chongqing University Cancer Hospital, which included 48 IRP cases and 142 non-IRP cases. The screening flow is illustrated in **Figure 2**.

The median ages of IRP and non-IRP patients were 61.00 [IQR: 54.75–67.00] years and 58.00 [IQR: 52.00, 67.00] years, respectively. The number of males was higher than that of females in both the groups. The baseline body temperature showed a statistical difference between the IRP and non-IRP groups (the distribution features are provided in **Supplemental Appendix 1**; **Figure S1**). The most common stage of cancer was stage IV, followed by stage III in all cohorts. Karnofsky performance status (KPS) score was median in 80. The relationship between these factors and the occurrence of IRP are further screened in the following sections. We did not find significant differences in underlying diseases (hypertension, diabetes, CHD, viral hepatitis, and lung-related diseases) between the IRP and non-IRP groups. However, the number of underlying diseases was statistically significant (*P <*0.05). IRP risk was statistically different in patients treated with PD-1 and PD-L1. Patients in the PD-1 group were less likely to develop IRP than those in the PD-L1 group. Moreover, the combination of non-antitumor drugs, history of radiation therapy, T lymphocyte count, and percentage of basophils might have contributed to IRP outcome (*P <*0.05) (**Table 1**).

**Table 1:** The results of characteristics of patients by univariate analysis.

	IRP (n = 48)	Non-IRP (N = 142)	*P-*value
Sex			0.331
Male	41 (85.4)	110 (77.5)	
Female	7 (14.6)	32 (22.5)	
Age (y)	61.00 [54.75, 67.00]	58.00 [52.00, 67.00]	0.358
BMI	22.98 [20.82, 25.38]	23.56 [21.48, 25.08]	0.592
Body Temperature (°C)	36.60 [36.50, 36.80]	36.50 [36.30, 36.70]	**0.045**
Systolic blood pressure	121.50 [108.25, 129.00]	124.00 [112.25, 133.75]	0.346
Diastolic blood pressure	77.00 [70.00, 83.75]	79.50 [71.00, 85.00]	0.442
Smoking (yes)	31 (64.6)	72 (50.7)	0.133
Drinking (yes)	13 (27.1)	27 (19.0)	0.327
KPS score			0.169
≤70	17 (35.4)	31 (21.8)	
80	88 (62.0)	24 (50.0)	
≥90	7 (14.6)	23 (16.2)	
Cancer stage			0.708
I	0 (0.0)	1 (0.7)	
II	1 (2.1)	3 (2.1)	
III	18 (37.5)	42 (29.8)	
IV	29 (60.4)	95 (67.4)	
Cancer category			0.013
NSCLC	42 (87.5)	96 (67.6)	
Non NSCLC^1^	6	46	
Type of underlying diseases			
Hypertension	11 (22.9)	20 (14.1)	0.228
Diabetes	10 (20.8)	16 (11.3)	0.154
Coronary Heart Disease	4 (8.3)	7 (4.9)	0.474
Viral Hepatitis	3 (6.2)	13 (9.2)	0.765
Lung-related disease	8 (16.7)	18 (12.7)	0.651
Number of underlying diseases			**<0.001**
0	6 (12.5)	62 (43.7)	
1	15 (31.2)	52 (36.6)	
≥2	27 (56.2)	28 (19.7)	
History of lung diseases	2 (4.2)	35 (32.7)	**0.004**
ICI drugs			**0.047**
PD-L1	5 (10.4)	4 (2.8)	
PD-1	43 (89.6)	138 (97.2)	
ICIs drugs			**<0.001**
Attilizumab	2 (4.2)	3 (2.1)	
Carrilizumab	19 (39.6)	36 (25.4)	
Tirelizumab	3 (6.2)	31 (21.8)	
Nevirumab	2 (4.2)	6 (4.2)	
Perbolizumab	11 (22.9)	12 (8.5)	
Toripalimab	5 (10.4)	18 (12.7)	
Sindillizumab	0 (0.0)	33 (23.2)	
others	6 (12.5)	2 (1.4)	
ICIs drug dosage (mg)	200.00 [200.00, 200.00]	200.00 [200.00, 200.00]	0.158
First time for immunotherapy (yes)	45 (93.8)	129 (90.8)	0.765
Course of cancer treatment	4.00 [3.00, 7.00]	5.00 [3.00, 7.00]	0.222
Number of other antitumor drugs			0.153
0	8 (17.0)	35 (24.6)	
1	14 (29.8)	21 (14.8)	
2	24 (51.1)	83 (58.5)	
3	1 (2.1)	2 (1.4)	
≥4	0 (0.0)	1 (0.7)	
History of other antitumor drugs exposure (yes)	39 (83.0)	107 (75.4)	0.546
Number of non-antitumor drugs			**0.027**
0	13 (50.0)	102 (74.5)	
1	3 (11.5)	17 (12.4)	
2	4 (15.4)	7 (5.1)	
3	3 (11.5)	5 (3.6)	
4	2 (7.7)	3 (2.2)	
5	1 (3.8)	1 (0.7)	
≥6	0 (0.0)	2 (1.5)	
History of non-antitumor drugs exposure (yes)	13 (50)	35 (25.5)	**0.005**
Surgery (yes)	11 (26.2)	42 (29.6)	0.817
History of radiation therapy (yes)	27 (64.3)	48 (33.8)	**0.002**
History of chemotherapy (yes)	32 (66.7)	78 (54.9)	0.210
Number of previous anti-tumor drugs			0.070
0	17 (35.4)	53 (40.2)	
1	1 (2.1)	3 (2.3)	
2	28 (58.3)	51 (38.6)	
3	2 (4.2)	23 (17.4)	
4	0 (0.0)	1 (0.8)	
≥5	0 (0.0)	1 (0.8)	
History of anti-tumor drugs exposure (yes)	31 (64.6)	89 (62.7)	0.866
CD4^+^ lymphocyte count	264.50 [186.75, 490.00]	403.50 [256.25, 582.75]	0.054
Percentage of CD4^+^ lymphocytes	31.05 [25.62, 41.05]	35.20 [28.33, 44.88]	0.102
CD8^+^ lymphocyte count	260.50 [189.50, 356.75]	308.00 [183.75, 406.50]	0.249
Percentage of CD8^+^ lymphocytes	28.20 [21.52, 37.38]	26.75 [20.52, 33.22]	0.565
T lymphocyte count	570.00 [427.50, 867.25]	752.00 [554.50, 1049.50]	**0.049**
Percentage of T lymphocytes	67.60 [58.80, 75.55]	71.10 [62.00, 77.35]	0.292
B lymphocyte count	77.50 [31.75, 129.75]	90.00 [53.50, 141.50]	0.331
Percentage of B lymphocytes	7.90 [4.65, 13.22]	8.50 [5.35, 12.85]	0.629
NK cell count	201.50 [124.00, 297.25]	206.00 [122.00, 289.50]	0.896
Percentage of NK cell	18.95 [14.78, 31.52]	18.60 [12.45, 27.85]	0.488
Red blood cell	3.92 [3.38, 4.34]	3.84 [3.40, 4.17]	0.592
Hemoglobin	118.50 [103.75, 127.00]	116.50 [106.75, 129.00]	0.781
Hemameba	5.72 [4.30, 7.91]	5.17 [4.15, 6.56]	0.139
Percentage of lymphocytes	17.75 [11.18, 24.52]	20.25 [13.30, 27.77]	0.151
Percentage of monpcytes	9.20 [6.00, 12.35]	10.05 [7.60, 12.50]	0.370
Percentage of neutrophilic granulocyte	68.70 [59.65, 79.20]	66.40 [58.43, 74.35]	0.278
Percentage of eosinophils	1.00 [0.30, 3.80]	1.90 [0.60, 3.15]	0.379
Percentage of basophils	0.30 [0.10, 0.60]	0.40 [0.30, 0.70]	**0.034**
Blood platelet	184.00 [144.25, 256.50]	181.00 [139.50, 233.75]	0.354

1: non-NSCLC contains malignant melanoma, small cell lung cancer, nasopharyngeal carcinoma, cervical cancer, Hodgkin’s lymphoma, ovarian cancer, diffuse large B-cell lymphoma, esophageal carcinoma, gastric cancer, bladder cancer and others. The bold values means less than 0.05.

### 3.2 IRP risk prediction

Eighteen variables (a list can be found in **Table 1**) were found associated with ICI-related IRP in the univariate analyses. Under the repeated cross-validation framework, we tuned the hyperparameters and finally determined that the model with an alpha of 1.000 and lambda of 0.026 generated the best model performance. The final prediction model was trained on the full training set by using these parameters. The final prediction model included 11 predictors: sindillizumab, ≥2 underlying diseases, history of lung diseases, tirelizumab, NSCLC, percentage of CD4^+^ lymphocytes, body temperature, KPS score ≤70, hemoglobin, cancer stage IV, and history of antitumor therapy. The coefficients of the 11 predictors are presented in **Supplemental Appendix 3** (**Table S5**). An online ICI-related IRP risk calculator was developed using our final predicted model and can be accessed through https://lin-yu.shinyapps.io/IRPcalculator/.

#### 3.2.1 Model performance

The final prediction model had adequate discrimination, with a cross-validated AUC of 0.81 (95% CI: 0.79–0.84) over the validation sets. The AP value was considerably higher than the event rate (AP = 0.58; event rate = 25%), and the Spiegelhalter-z was 0.34 (P-value: 0.74), indicating good calibration. A predictive model was applied to the test set for external validation. The AUC estimate was 0.81 (95% CI: 0.55–0.90), the AP was 0.68, and the Spiegelhalter-z was −0.29 (P-value: 0.77) (**Table 2**). Therefore, we conclude that our predictive model has the potential for IRP risk prediction.

**Table 2:** Summary of performance of model on training and test datasets.

Data	AUC (95% CI)	AP	SBrS	Spiegelhalter-z (*p*-value)
Validation^1^	0.81 (0.79–0.84)	0.58	0.27	0.34 (0.74)
Test^2^	0.81 (0.55–0.90)	0.68	0.30	−0.29 (0.77)

^1^Validation data refers to thirty leave-out parts in repeated CV framework. ^2^Test data is the 20% of the whole study subjects which was used to mimic an external data source for external validation.

#### 3.2.2 Variable importance

**Figure 3:**
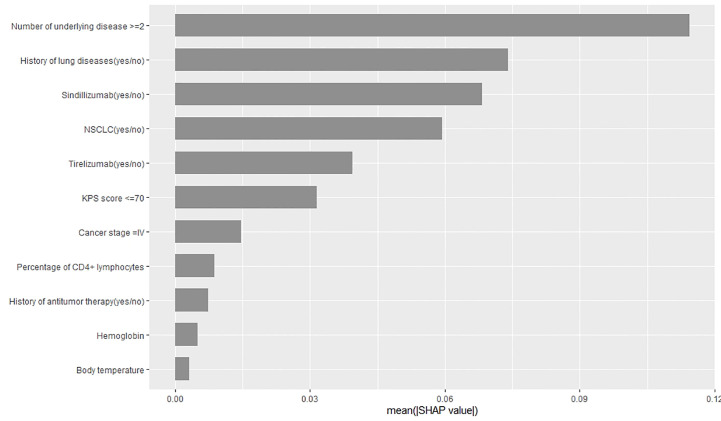
The rank of features in the prediction model by the degree of importance.

**Figure 4:**
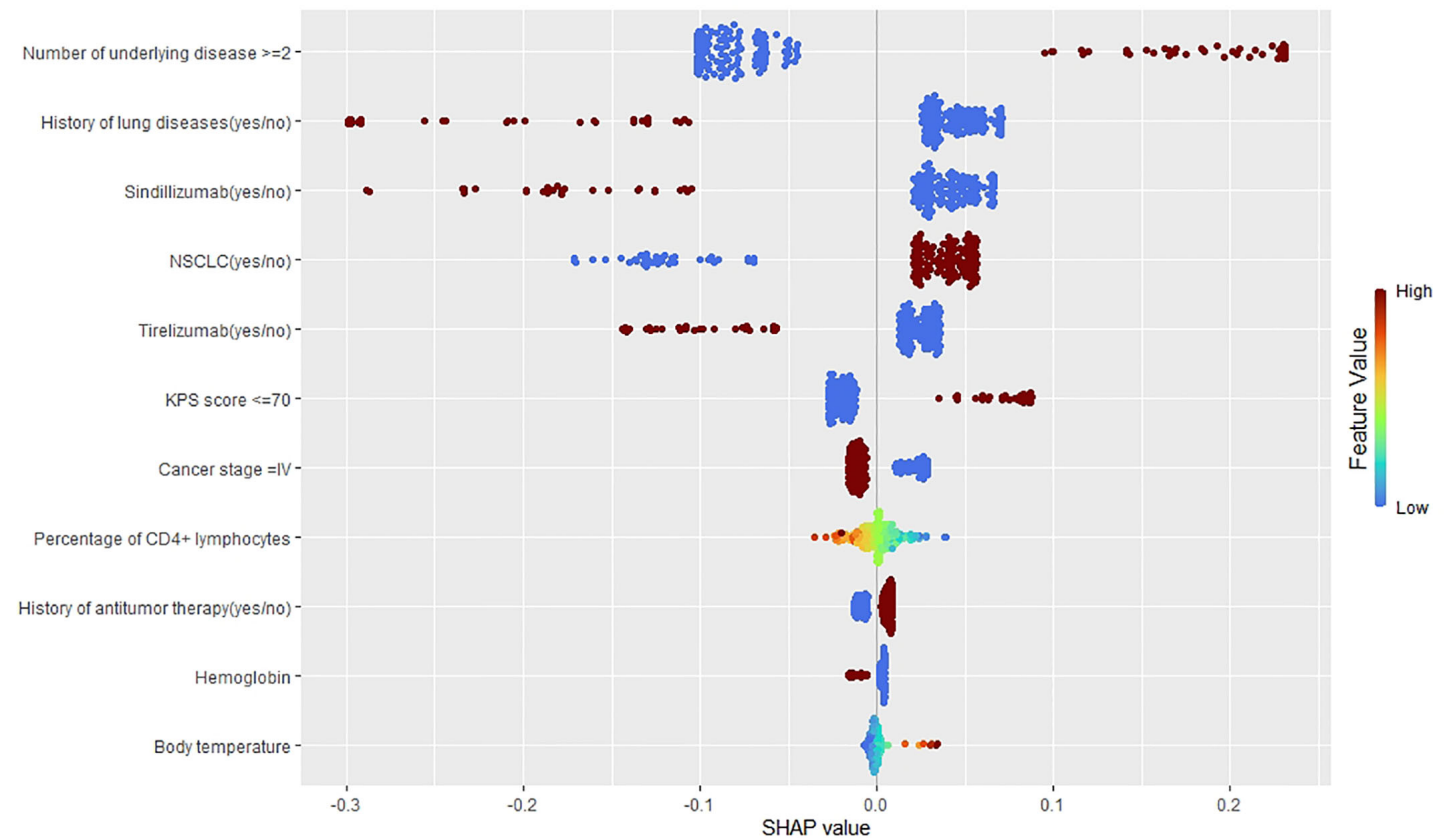
The SHAP value of features in the prediction model.

We used feature importance and SHAP plots to visualize the variable importance (**Figure 3**). Predictors are shown in order of global feature importance, with the first being the most important and the last being the least important. We also used the SHAP value to visualize the variable importance and direction of the association (**Figure 4**). A positive SHAP value indicates a positive association between predictors and ICI-related IRP; likewise, a negative SHAP value corresponds to a negative association between predictors and ICI-related IRP. The SHAP plot indicated that the number of underlying diseases ≥2, NSCLC, KPS score ≤70, history of antitumor therapy, other ICI drugs, and body temperature were positively associated with IRP, whereas the remaining predictors were negatively related to IRP.

#### 3.2.3 Calibration plots

**Figure 5:**
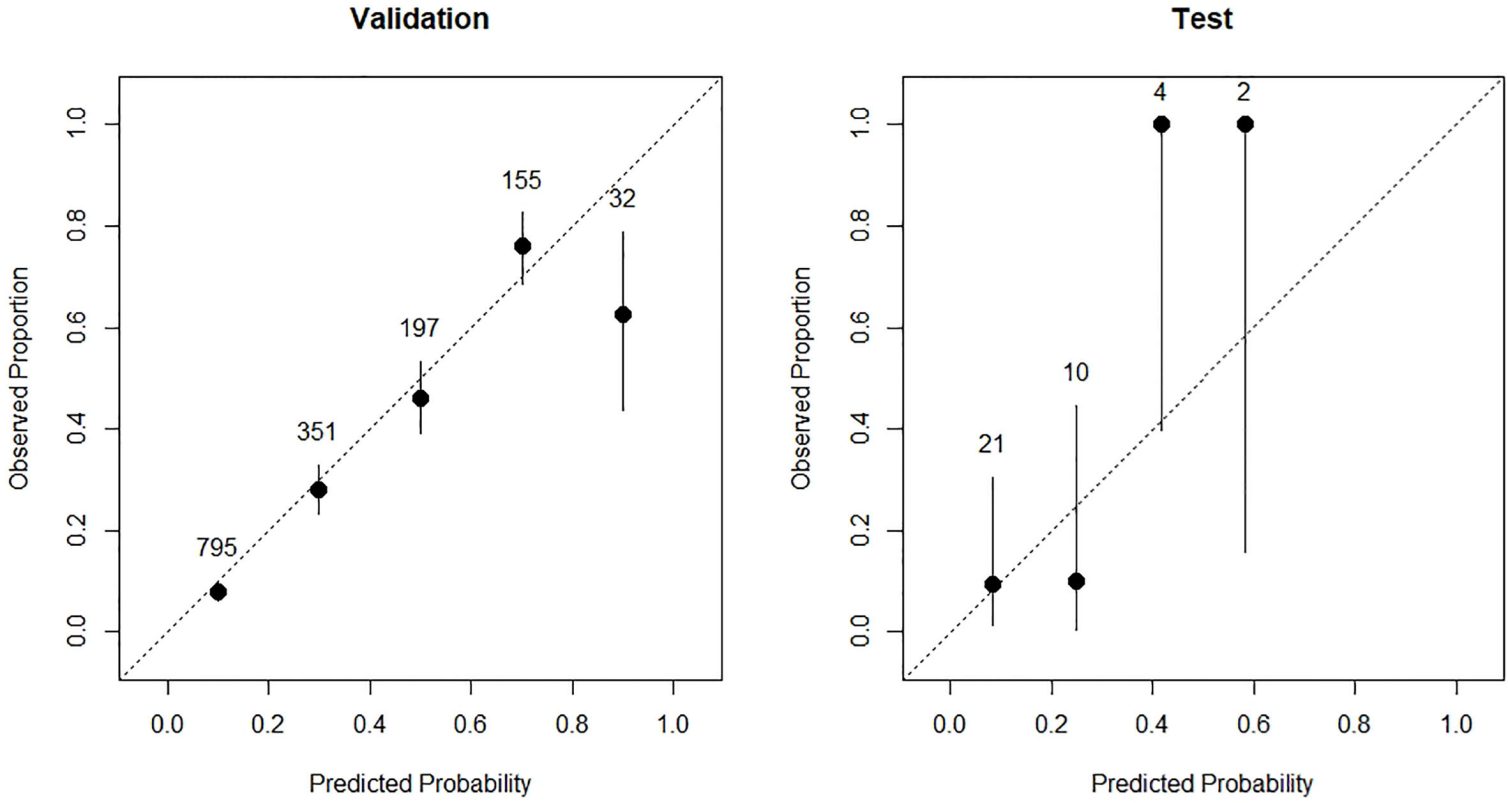
The calibration plots of validation cohort (left) and test cohort (right) of the prediction model.

Calibration plots were used to visualize the calibration (**Figure 5**). In the validation set, we observed great calibration for patients with predicted risk less than 0.8, and overestimated IRP risk in patients with predicted risk between 0.8 and 1.0, respectively. In the test set, we found that the model underestimated the risk in high IRP risk group and overestimated the IRP risk in the low-risk group. Taken together, the predicted probability risks in the subgroups were close to the observed proportion, suggesting that our model was well-calibrated.

#### 3.2.4 Risk stratification

Using 5%, 20%, 50%, and 80% as cut-offs, the predicted probabilities of IRP were stratified into four risk categories: <5%, 5% to <20%, 20% to <50%, 50% to 80%, and ≥80%, each corresponding to a different level of risk, including low-, medium-low, medium, median-high, and high-risk groups. **Table 3** shows that our model performed well with regard to the risk stratification. In the validation set, among 31 participants with a predicted IRP risk greater than 80%, 61% (19 out of 31) developed IRP; 325 participants with a predicted IRP risk of less than 0.05, 5% (15/325) of them developed IRP. Risk stratification in the test set indicated good calibration. Two and five of the 17 and 13 individuals who were predicted to be at medium-low and medium IRP risk, respectively, developed IRP.

**Table 3:** The risk stratification of ICIs-related IRP in our prediction model.

	Validation		Test	
Risk groups	# of IRP event/ # of patients	PPV	# of IRP event/ # of patients	PPV
0.05 (low-risk)	15/325	0.05	0/5	0.00
5%–20% (medium-low)	48/466	0.10	2/17	0.12
20%–50% (medium)	146/470	0.31	5/13	0.38
50%–80% (medium-high)	162/238	0.68	2/2	1.00
>80% (high-risk)	19/31	0.61	0/0	/

PPV, positive predictive value; samples are 10 times that of the original training set, due to the repeated CV framework (repeat = 10). "#" means number and "/" means division sign.

## 4 Discussion

The utility of electronic medical records (EMR) has expanded from data storage to data utilization using various methods, which could guide clinical decisions and predict important outcomes (15). Establishing an ICI-related IRP prediction model using EMR and machine learning algorithms is an effective and low-cost approach. We identified potential IRP predictors such as the number of underlying diseases, ICI drugs (sindilizumab and tirelizumab), history of lung disease, NSCLC, percentage of CD4^+^ lymphocytes, and body temperature, KPS score ≤70, hemoglobin, cancer stage IV, and history of antitumor therapy. We also developed and validated an IRP prediction model for patients with cancer, using the Elastic Net model. We further applied the final model to establish a user-friendly IRP risk calculation tool, in which personalized IRP risk could be calculated using relevant clinical information.

We found that the total number of underlying diseases was the most important risk factor for IRP. Patients with more than two underlying diseases might have a greater risk of developing IRP if they received ICI treatment. This phenomenon might contribute to the poor performance status (16). In our study, we found that hypertension, diabetes, coronary heart disease, viral hepatitis, and chronic obstructive pulmonary disease were the most common combination of diseases. Interestingly, we did not find that these combination diseases had statistically significant differences associated with IRP, which differs from previous studies. Some previous studies have suggested that pre-existing lung diseases, such as chronic obstructive pulmonary disease, might contribute to IRP, but this was not confirmed by a statistically significant difference (7, 11, 17).

The incidence of IRP is affected by anti-PD-1 agents. In contrast to anti-PD-L1 and anti-CTLA-4 drugs, anti-PD-1 agents are more likely to cause adverse reactions in the lungs (7). However, we could not rule out the possibility that other ICIs could result in IRP in patients who underwent immunotherapy. In our study, we did not include data on combination immunotherapy. Previous studies have suggested that patients receiving combination immunotherapy are associated with a higher incidence of lung toxicity than patients receiving monotherapy (14, 18). Combination therapy with other antitumor drugs, such as chemotherapy, was the most common treatment, and there was no statistically significant difference between IRP and non-IRP. There is still limited evidence on the IRP of ICI combined with chemotherapy (19).

NSCLC was a risk factor for IRP, which was more common in lung cancer patients treated with ICIs than in patients with other cancers. Some studies have demonstrated that IRP has been repeatedly reported in NSCLC patients compared to patients diagnosed with melanoma and head and neck squamous cell carcinoma (17, 20). However, the biological mechanism of IRP in NSCLC is poorly understood. Dysregulated activation of T cells in peripheral lung tissue (21) and the predisposition of peritumoral lung tissue to irAEs (7) may play an important role.

For laboratory indexes, CD4^+^ lymphocyte count after ICI treatment was negatively correlated with IRP; that is, a smaller CD4^+^ lymphocyte count is related to higher IRP risk. CD4^+^ lymphocytes are a subpopulation of T lymphocytes, and CD4^+^ cell can cooperate with cytotoxic T lymphocytes contributing to the efficacy of immunotherapy (22). ICIs may increase the greater magnitude of T-cell proliferation or decrease CD4^+^ cell-mediated immunosuppression (23). A lower count of CD4^+^ cells may indicate an active immune response. IRP is an active inflammatory infiltrative lung disease associated with an immune response (8), and the count of CD4^+^ lymphocyte cells can predict the risk of IRP.

In this study, we established a prediction model for the IRP of cancer patients using ICIs. Accordingly, an online calculation tool was developed. Users can upload relevant information to obtain the IRP risk immediately. An early understanding of the risks of IRP will improve the clinical benefit for patients. However, this study had some limitations. First, this was a retrospective study with a lack of prospective verification. This may introduce selection bias, specifically sampling bias, which may limit the generalizability of the results. In addition, the data selected from a single resource may not be representative of the characteristics of the general population. Therefore, the external validation in this study did not guarantee a good performance when applying the model to the general population.

Second, the small sample size may have caused imbalanced distributions of variables and biased the estimate of IRP risk. In our study dataset, we observed skewed distributions for sex and PD-1 therapy, where female patients and patients who underwent PD-L1 therapy were underrepresented. Our analysis revealed that there was no statistically significant difference in the sex distributions between the IRP and non-IRP groups and that sex was not a predictor of IRP, which is consistent with earlier meta-analyses and the findings of a real-world study (24). Although the proportion of patients who received PD-1 therapy in two comparison groups was statistically significant, PD-1 therapy was not a predictor of IRP risk. Interpretation of the observed statistical significance should be done with care, as the PD-1 therapy distribution in our study sample could be imbalanced and not representative of the true pattern of the data. To address this concern, we used a repeated CV framework to make full use of the data; however, the observed model performance in the test set should be interpreted with caution because of the limited sample size. Third, variables (such as Tumor Mutational Burden (25)) that were found to be associated with IRP could not be included in the prediction model because of the high missing rates. In the future, a higher-quality data, multicenter, larger sample, and prospective study is needed to optimize and prove the validity of the IRP prediction model before it can be used in a clinical setting.

## Data availability statement

The original contributions presented in the study are included in the article/**Supplementary material**. Further inquiries can be directed to the corresponding authors.

## Author contributions

LG, JG, XS, LY, XC, and Y-SL contributed to conception and design of the study. LG and JG screened the data and organized the database. JG, XS, and LY performed the statistical analysis. LG, JG, XS, LY, and BL wrote the first draft of the manuscript. Y-SL and XC reviewed and modified the manuscript. All authors contributed to the article and approved the submitted version.
