# Supplementary material for: Identification and prediction of immune checkpoint inhibitors-related pneumonitis by machine learning
Source: Front Immunol. 2023 Jun 29;14:1138489. doi: 10.3389/fimmu.2023.1138489 (PMC10338954; doi:10.3389/fimmu.2023.1138489)
Supplement: Supplementary file 1 [file DataSheet_1.pdf]

Supplementary Material

Identification and prediction of immune checkpoint inhibitors-related pneumonitis by machine learning

Li Gong[1†], Jun Gong[2†], Xin Sun[3], Lin Yu[3], Bin Liao[1], Xia Chen[4*], Yong-sheng Li[1*]

*** Correspondence:**

Xia Chen (kathleentj@cqu.edu.cn)

Yongsheng Li (lys@cqu.edu.cn)

1 Supplemental Appendix 1

Table S1. Data dictionary of variables.

	Variable name	Type(units)	Description
outcome	IRP	categorical/factor	IRP status (2 levels): *IRP, Non-IRP*
demographic	Sex		biological sex (2 levels) : *Male, Female*
	Age(y)	numeric (years)	age >=18
	BMI	numeric	
	Body Temperature	numeric(℃)	
	Systolic blood pressure	numeric	
	Diastolic blood pressure	numeric	
	Smoking (yes)	categorical/factor	
	Drinking (yes)	categorical/factor	
	KPS score	numeric	
	Cancer stage	categorical/factor	(4 levels): I，II，III，IV
	Number of underlying diseases		
	History of lung diseases		

Treatment	ICIs drugs		(8 levels): *Attilizumab,Carrilizumab, Tirelizumab,Nevirumab, Perbolizumab, Toripalimab,Sindillizumab, others*
	ICIs drug dosage	numeric(mg)	
	First time for immunotherapy	categorical/factor	(2 levels): *yes, no*
	Course of cancer treatment	count	
	Number of other antitumor drugs	count	
	Number of non-antitumor drugs	count	
	Surgery	categorical/factor	(2 levels): *yes, no*
	History of radiation therapy	categorical/factor	(2 levels): *yes, no*
	History of chemotherapy	categorical/factor	(2 levels): *yes, no*
	Number of previous anti-tumor drugs	count	
lab results	$CD4^+$ lymphocyte count	numeric	
	Percentage of $CD4^+$ lymphocytes	numeric	
	$CD8^+$ lymphocyte count	numeric	
	Percentage of $CD8^+$ lymphocytes	numeric	
	T lymphocyte count	numeric	
	Percentage of T lymphocytes	numeric	
	B lymphocyte count	numeric	
	Percentage of B lymphocytes	numeric	
	NK cell count	numeric	
	Percentage of NK cell	numeric	
	Red blood cell	numeric	
	Hemoglobin	numeric	
	Hemameba	numeric	
	Percentage of lymphocytes	numeric	
	Percentage of monpcytes	numeric	
	Percentage of neutrophilic granulocyte	numeric	
	Percentage of eosinophils	numeric	

	Percentage of basophils	numeric	
	Blood platelet	numeric	

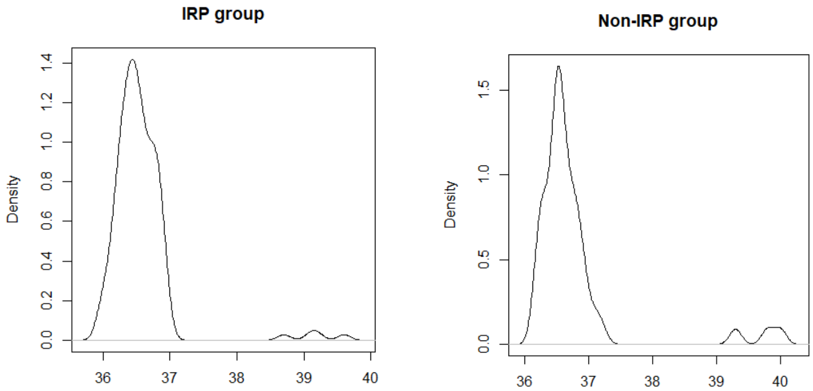

Figure S1. The body temperature distributions between IRP and non-IRP groups

Table S2. Baseline variable comparisons.

	**IRP (n=48)**	**Non-IRP (N=142)**	**P value**
$CD4^+$ lymphocyte count	402.50 [269.00, 624.75]	462.50 [292.50, 673.75]	0.494
Percentage of $CD4^+$ lymphocytes	34.45 [28.33, 41.30]	37.10 [28.90, 45.30]	0.250
$CD8^+$ lymphocyte count	343.00 [191.50, 439.25]	300.50 [228.00, 432.50]	0.631
Percentage of $CD8^+$ lymphocytes	25.75 [20.68, 33.00]	25.05 [20.00, 33.85]	0.630
T lymphocyte count	799.50 [559.75, 1160.50]	830.00 [592.00, 1134.00]	0.908
Percentage of T lymphocytes	67.55 [60.08, 78.30]	69.80 [59.90, 77.20]	0.528
B lymphocyte count	99.00 [57.75, 185.00]	130.00 [66.00, 197.00]	0.464
Percentage of B lymphocytes	8.85 [5.93, 13.15]	9.30 [6.50, 13.50]	0.491

NK cell count	218.00 [157.00, 330.00]	236.00 [127.00, 339.00]	0.829
Percentage of NK cell	20.50 [11.80, 32.80]	19.30 [13.00, 24.40]	0.298
Interleukin-2	565.00 [487.00, 654.00]	477.50 [385.00, 685.75]	0.355
Interleukin-6	3.98 [2.53, 12.00]	7.18 [4.70, 14.83]	0.163
Interleukin-8	16.00 [12.40, 21.90]	29.65 [13.03, 91.62]	0.067
Interleukin-10	5.00 [5.00, 5.00]	5.00 [5.00, 5.00]	0.350
Tumor necrosis factor α	9.33 [8.50, 10.60]	9.32 [7.39, 14.05]	0.865
Red blood cell	4.20 [3.85, 4.72]	4.11 [3.59, 4.56]	0.357
Hemoglobin	127.00 [113.00, 138.00]	123.00 [111.00, 140.00]	0.903
Hemameba	5.92 [5.14, 7.07]	6.15 [4.80, 7.85]	0.689
Percentage of lymphocytes	19.20 [12.30, 23.90]	20.30 [15.20, 25.80]	0.277
Percentage of monpcytes	9.10 [7.20, 11.70]	8.20 [6.65, 11.15]	0.355
Percentage of neutrophilic granulocyte	67.30 [59.00, 81.50]	67.30 [60.30, 72.75]	0.604
Percentage of eosinophils	1.60 [0.60, 3.60]	2.10 [1.25, 3.50]	0.134
Percentage of basophils	0.50 [0.20, 0.60]	0.50 [0.30, 0.70]	0.09
Blood platelet	226.00 [186.00, 279.00]	213.00 [162.00, 286.50]	0.961
Partial pressure of carbon dioxide	42.00 [39.25, 44.00]	44.00 [41.00, 48.00]	**0.009**
Oxygen partial pressure	83.50 [74.75, 98.75]	79.00 [72.00, 89.00]	0.170

HCO	25.35 [22.73, 27.12]	26.60 [25.40, 28.80]	0.002
SBC	24.90 [23.80, 26.40]	26.30 [25.10, 27.30]	0.015
Oxyhemoglobin saturation	97.00 [97.00, 97.00]	95.00 [94.00, 97.00]	0.416
BE	-1.20 [-1.20, -1.20]	1.90 [0.20, 3.10]	0.139
Whole blood lactic acid	1.30 [1.30, 1.30]	1.90 [1.50, 2.40]	0.245
PH	7.40 [7.37, 7.43]	7.40 [7.37, 7.42]	0.494

2 Supplemental appendix 2

Table S3. A summary of missingness.

No.	Variable Name	Count	Percentage (%)
1	TMB	176	91.67
2	PH value	141	73.44
3	BE	138	71.88
4	Whole blood lactic acid	138	71.88
5	Partial pressure of carbon dioxide	119	61.98
6	Interleukin-2	110	57.29
7	Oxygen partial pressure	101	52.60
8	HCO	101	52.60
9	SBC	101	52.60
10	Oxyhemoglobin saturation	101	52.60
11	Interleukin-6	100	52.08
12	Interleukin-8	100	52.08
13	Interleukin-10	100	52.08
14	Tumor necrosis factor α	100	52.08
15	$CD4^+$ lymphocyte count(baseline)	31	16.15
16	Number of non-antitumor drugs	27	14.21
17	CD8 lymphocyte count	24	12.50
18	Percentage of $CD8^+$ lymphocytes	24	12.50

19	$CD4^{+}$ lymphocyte count	24	12.50
20	T lymphocyte count	23	11.98
21	Percentage of T lymphocytes	23	11.98
22	B lymphocyte count	23	11.98
23	Percentage of B lymphocytes	23	11.98
24	NK cell count	23	11.98
25	Percentage of NK cell	23	11.98
26	Red blood cell	13	6.77
27	ICIs drug dosage (mg)	12	6.25
28	Number of previous anti-tumor drugs	10	5.26
29	Hemoglobin	8	4.17
30	Hemameba	8	4.17
31	Percentage of lymphocytes	8	4.17
32	Percentage of monpcytes	8	4.17
33	Percentage of neutrophilic granulocyte	8	4.17
34	Percentage of eosinophils	8	4.17
35	Percentage of basophils	8	4.17
36	Blood platelet	8	4.17
37	Surgery	6	3.12
38	History of radiation therapy	6	3.12
39	Course of cancer treatment	4	2.08
40	BMI	3	1.56
41	Systolic blood pressure	2	1.04
42	Diastolic blood pressure	2	1.04
43	KPS score	2	1.04
44	Temperature (℃)	1	0.52
45	Cancer stage	1	0.52
46	Number of other antitumor drugs	1	0.52
47	Sex	0	0.00
48	Age(y)	0	0.00
49	Smoking	0	0.00
50	Drinking	0	0.00

51	Number of underlying diseases	0	0.00
52	History of lung diseases	0	0.00
53	ICIs drugs	0	0.00
54	First time for immunotherapy	0	0.00
55	History of chemotherapy	0	0.00

Note: variables with missingness larger than 15% were removed from the analysis.

3 Supplemental appendix 3

Modelling process

3.1 Pre-processing

Before modeling, we imputed missing data using the mode or median given none of the continuous predictors was normally distributed. we checked multicollinearity among the continuous candidate predictors using correlation (Pearson) function in R. we noticed five predictors with sparse distributions (shown in Figure S2), thus we grouped them to categorical variables, the mapping relationship is shown in Table 1. We also created dummy variables for categorical predictors with more than 2 levels.

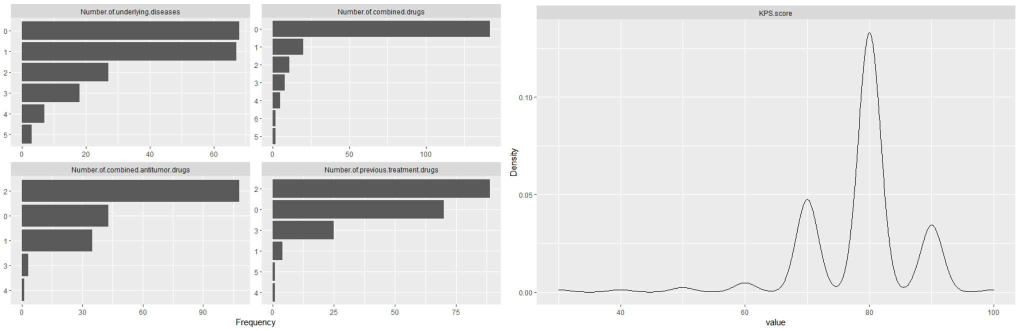

Figure S2. Sparse distributions of number of underlying disease, number of previous antitumor drugs, number of non-antitumor drugs, number of other antitumor drugs and KPS score.

Table S4. The categorical of predictors.

Predictor name	Original data type	Working data type
Number of underlying disease	count	categorical; 3 levels (0, 1, >=2)

Number of previous antitumor drugs	count	categorical; 2 levels (Yes/No)
Number of non-antitumor drugs	count	categorical; 2 levels (Yes/No)
Number of other antitumor drugs	count	categorical; 2 levels (Yes/No)
KPS score	continuous	categorical; 3 levels (<=70; 80; >=90)

3.2 Training-validation-test framework

We used stratified sampling to divide the working dataset into two parts: training and test sets (with a ratio of 8:2), where the test set is used to mimic an external data for external validation. The training set will be further divided into training and validation sets using cross-validation framework to allow interval validation.

3.3 Modelling

Risk prediction models were built using the training set. Specifically, the training set was randomly partitioned into three roughly equal size parts, we then left out one part as the validation set and model was built on the remaining parts. The leave-out-modelling process was conducted recursively until each part was treated as validation set for once. The cross-validation modelling process was repeated for 10 times. Therefore, the number of training samples is 10 times that of the original training set. We Tuned one hundred combinations of hyperparameters, where we specified ten different alphas: 0.1,0.2,0.3, 0.4,…,1.0, and ten different lambdas: 0.000171526, 0.000396247, 0.000915382, 0.002114651, 0.004885118, 0.011285256, 0.026070404, 0.060226016, 0.139129907.

3.4 Performance Evaluation

Performance matrices including Scaled brier score, AUC, AP and Spiegelhalter-z statistics were computed for the validation and test sets respectively. The confidence interval of AUC was computed using bootstrapping method.

Table S5 Intercept coefficients of final prediction model

Variable name	Coefficient
Intercept	-1.474
KPS score <=70	0.530
Cancer stage =IV	-0.196
History of antitumor therapy(yes/no)	0.048
Percentage of CD4+ lymphocytes	-0.011
Hemoglobin	-0.011
NSCLC(yes/no)	1.265
Body temperature	0.027
History of lung diseases(yes/no)	-2.310

Tirelizumab(yes/no)	-1.036
Sindillizumab(yes/no)	-2.122
Number of underlying disease >=2	1.690
